# Uptake of polyphosphate microparticles *in vitro* (SaOS-2 and HUVEC cells) followed by an increase of the intracellular ATP pool size

**DOI:** 10.1371/journal.pone.0188977

**Published:** 2017-12-29

**Authors:** Werner E. G. Müller, Shunfeng Wang, Matthias Wiens, Meik Neufurth, Maximilian Ackermann, Dinko Relkovic, Maria Kokkinopoulou, Qingling Feng, Heinz C. Schröder, Xiaohong Wang

**Affiliations:** 1 ERC Advanced Investigator Grant Research Group at the Institute for Physiological Chemistry, University Medical Center of the Johannes Gutenberg University, Mainz, Duesbergweg 6, Mainz, Germany; 2 Institute of Functional and Clinical Anatomy, University Medical Center of the Johannes Gutenberg University, Mainz, Germany; 3 Fidelta Ltd., Prilaz baruna Filipovića 29, Zagreb, Croatia; 4 Max Planck Institute for Polymer Research, Ackermannweg 10, Mainz, Germany; and; 5 Key Laboratory of Advanced Materials of Ministry of Education of China, School of Materials Science and Engineering, Tsinghua University, Beijing, China; Cedars-Sinai Medical Center, UNITED STATES

## Abstract

Recently two approaches were reported that addressed a vitally important problem in regenerative medicine, i. e. the successful treatment of wounds even under diabetic conditions. Accordingly, these studies with diabetic rabbits [Sarojini et al. PLoS One 2017, 12(4):e0174899] and diabetic mice [Müller et al. Polymers 2017, 9, 300] identified a novel (potential) target for the acceleration of wound healing in diabetes. Both studies propose a raise of the intracellular metabolic energy status *via* exogenous administration either of ATP, encapsulated into lipid vesicles, or of polyphosphate (polyP) micro-/nanoparticles. Recently this physiological polymer, polyP, was found to release metabolic energy in form of ATP into both the extra- and also intra-cellular space. In the present work the uptake mechanism of the amorphous polyP microparticles “Ca-polyP-MP” has been described and found to be a clathrin-dependent endocytosis import, based on inhibition studies with the inhibitor trifluoperazine, which blocks the clathrin-dependent endocytosis import. The experiments had been performed with SaOS-2 cells, by studying the uptake and distribution of the electron-dense particles into the cells, and with HUVEC cells, for analysis of the intracellular accumulation of polyP, visualized by fluorescent staining of polyP. Concurrently with the uptake of particular polyP the intracellular ATP level increased as well. In contrast to “Ca-polyP-MP” the soluble polyP, administered as “Na-polyP[Ca^2+^]”, did not cause an increase in the intracellular Ca^2+^ level, suggesting a different mode of action of these two forms of polyP. Based on existing data on the effect of polyP and ATP on the induction of vascularization during wound repair, both groups (Sarojini et al. and Müller et al.) propose that the acceleration of wound repair is based on an increased metabolic energy supply directly to the regenerating wound area.

## Introduction

ATP is the source of energy for (almost) any kind of intracellular metabolic (anabolic) syntheses [1–3]. Based on recent experimental data, polyphosphate (polyP) has been proposed as a storage for the extra- and intracellular supply of metabolic energy [4,5]. This physiological polymer exists in large amounts both intra- and extracellularly also in mammalian cells (reviewed in: [6]). During hydrolytic cleavage of polyP with the enzyme alkaline phosphatase (ALP), energy-rich phosphoanhydride bonds are cleaved under a release of free energy (ΔG°) of about -38 kJ mol^-1^ [with respect to 1 mole of anhydride bonds; at pH 5 [7]. Extracellularly, this metabolic energy might be utilized/transferred, at least partially, for the formation of energy-rich phosphoanhydride bonds in ADP and ATP, mediated by the ALP and the adenylate kinase (AK) [5]. Since these enzymes occur not only extracellularly but also intracellularly [8,9], it appears feasible that an energy transfer from polyP to AMP/ADP after ALP hydrolysis can also occur intracellularly *via* this route from polyP to ADP/ATP. However, polyP in the soluble (non-particulate) dissociated ionic form can only hardly penetrate the cell membrane (see: [10]). Under physiological conditions, polyP is formed intracellularly in the mitochondria [17] and, from there, is released into the cytosol [10]. Furthermore, in mammalian systems, polyP is present in large amounts in acidocalcisomes [11], and there is stored most likely as 200 nm-large microparticles, from where the polymer is released into the extracellular space. Previously we presented first experimental evidence that those particles are taken up by cells again *via* endocytosis [12,13].

If mammalian cells are exposed to polyP, formulated as amorphous nanoparticles/microparticles [14] similar to those present in the acidocalcisomes, they react with growth of the ATP pool [11]. Recently this physiological polymer polyP has been shown to accelerate tube formation by HUVEC cells if added extracellularly as Na^+^-salt [15]. Likewise we could demonstrate *in vivo* that polyP, if fabricated as nanoparticles [16], causes in mice a marked acceleration of wound healing, in particular in diabetic experimental animals [17]. Simultaneously and in parallel with our approaches it has been published that ATP, if delivered intracellularly after encapsulation into vesicles, induces a very rapid process of tissue regeneration in an experimental animal system (rabbits), which started already after 24 h [18]. This effect has even been demonstrated in diabetic animals [19] and is of clinical importance since it is well established that the energy supply in diabetic tissue is reduced and wound healing is impaired [20].

The finding that polyP, packed as amorphous nanoparticles/microparticles, can act as an intracellular reservoir for metabolic energy should be considered of pivotal impact for an amelioration/cure of many pathophysiological dysfunctions, such as wound healing [17] or neurodegeneration [21]. In the present study we describe the mechanism by which polyP particles are imported into the cells. The uptake of polyP microparticles was studied in two cells systems, SaOS-2 cells and HUVEC cells: SaOS-2 cells were exposed to polyP, then ultra-sectioned, and finally analysed by transmission electron microscopy, thus revealing electron-dense polyP particles. HUVEC cells were similarly incubated with the different polyP samples. In a second approach, polyP uptake was visualized by confocal laser scanning microscopy, after staining with DAPI according to established protocols [22–25]. The new data presented indicate that the polyP particles enter the cells through a clathrin-dependent endocytosis import mechanism. Furthermore experimental data strongly suggest that the cells react to polyP with an increased intracellular ATP pool.

It is discussed that the observed accelerated migration behavior, of e.g. endothelial cells during tube formation *in vitro* [15] or also during wound healing in experimental animal studies *in vivo* [17], is the result of the uptake and subsequent metabolic processing of polyP microparticles. The present study of polyP microparticles and the observed growth of the intracellular ATP pool also impressively confirms a recent study, where ATP (encapsulated into nano-/microparticles) initiated and maintained rapid tissue regeneration [18].

## Material and methods

### Materials

Na-polyphosphate (Na-polyP) with an average chain length of 40 phosphate units was from Chemische Fabrik Budenheim (Budenheim; Germany). For the experiments Na-polyP was complexed in a stoichiometric ratio (molar ratio) to Ca^2+^ of 2 [with respect to the phosphate monomer] to 1 Ca^2+^; abbreviated as “Na-polyP[Ca^2+^]” as described [26].

### Preparation of Ca-polyP *microparticles* and Ca-P particles

Amorphous Ca-polyP microparticles were prepared as outlined before [14]. To obtain this phase, the weight concentration ratio between Ca [as CaCl_2_] and P [as Na-polyP] was set to ≈2. In turn, 2.8 g of CaCl_2_ •·2 H_2_O (#223506; Sigma-Aldrich, Taufkirchen; Germany) were dissolved in 50 ml ethanol solution (96%) and added drop-wise to 1 g of Na-polyP, dissolved in 50 ml distilled water (room temperature; the pH was adjusted to 9–10). The suspension formed was kept at pH 10 (using 1 N NaOH) and stirred for 5 h. During this period microparticles assembled; they could be collected by filtration (Nalgene Filter Units [pore size 0.45 μm]; Cole-Parmer, Kehl/Rhein; Germany). Then, the sample was washed three times with ethanol and dried at 60°C. Subsequently, the material was sieved through a sieve shaker AS 200 (mesh size 100 μm; Retsch GmbH, Haan; Germany). These microparticles were termed “Ca-polyP-MP”.

In one control experiment “Ca-P particles” were prepared in the same way: 2.8 g of CaCl_2_ •·2 H_2_O were dropped into 1 g of Na-phosphate (Na_3_PO_4_; Sigma #342483). The resulting material that precipitated was filtrated and dried as described for polyP.

### Spectroscopic studies

X-ray diffraction (XRD) of the dried microparticle powder was performed with a Philips PW1820 diffractometer equipped with a monochromatic Cu-K_α_ radiation (λ = 1.5418 Å, 40 kV, 30 mA, 5 s, Δθ = 0.02), as described [27].

Energy dispersive X-ray (EDX) analysis was carried out with an EDAX Genesis EDX System attached to a scanning electron microscope (Nova 600 Nanolab; FEI, Eindhoven, The Netherlands) operating at 10 kV with a collection time of 30–45 s.

Fourier-transform infrared (FTIR) spectroscopy was conducted in an ATR-FTIR spectroscope/Varian 660-IR spectrometer (Agilent, Santa Clara; CA), equipped with a Golden Gate ATR unit (Specac, Orpington; UK). Each spectrum represents the average of 100 scans with a spectral resolution of 4 cm^-1^ (typically 550–1800 cm^-1^). A baseline correction, smoothing, and analysis of the spectra were achieved with the Varian 660-IR software package 5.2.0 (Agilent). The graphical display and annotation of the spectra were achieved with Origin Pro (version 8.5.1; OriginLab, Northampton; MA). The spectral peaks were assigned according to published data [14,28–31].

### Determination of the intracellular Ca^2+^ level

The intracellular Ca^2+^ level of HUVEC cells was quantified using the fluorescent emission method described [32]. In short, the subconfluent 2-d’s-old cultures were loaded with 10 μM Fura-2 AM (#F0888 Sigma-Aldrich) and, after washing with phosphate-buffered saline, exposed to the polyP sample, either “Na-polyP[Ca^2+^]” or “Ca-polyP-MP” (30 μg/mL, based on Na-polyP or the dried Ca-polyP microparticles), for up to 20 min. Then the signals (fluorescent emission at 510 nm) were recorded with a Cohu high-performance CCD camera (Cohu, Inc., Electronics Division; San Diego, CA), digitized and then stored. From these data the 340/380 excitation ratio (R340/380) was calculated. The values were normalized to 0.3 ratio units. The calcium “Imaging Calibration Kit” (Molecular Probes, Eugene; OR) was applied. One unit of fluorescence ratio 340/380 nm equals to 145 nM [Ca^2+^]_i_.

### Uptake studies with SaOS-2 cells

Human osteogenic sarcoma cells (SaOS-2 cells; [33]) have been purchased (Sigma #89050205). They were grown in McCoy’s medium (containing 1 mM CaCl_2_) with 5% heat-inactivated fetal calf serum (FCS), 2 mM l-glutamine and gentamicin (50 μg/mL) in six-well plates (Sigma-Greiner) as described [34]. The cells were seeded at a density of 2×10^4^ cells per 3 ml well and cultured for 3 d in medium/FCS. The cultures remained untreated (controls) or were treated for 3 d with 30 μg/mL of “Na-polyP[Ca^2+^]” or “Ca-polyP-MP”. Where indicated, the cells were pretreated (5 h) with 20 μM TFP (trifluoperazine dihydrochloride; #T8516 Sigma) to inhibit clathrin-dependent endocytosis [35].

After incubation the cells were harvested, fixed in paraformaldehyde/glutaraldehyde, embedded in LR-white resin (# 62661; Sigma-Aldrich). Finally ultrathin sections (80 nm) were prepared (Microsystems, Wetzlar; Germany) and contrasted with uranyl acetate and lead citrate as described previously [36]. The specimens were inspected with a transmission electron microscope [TEM].

For a semiquantitative determination of the number of microparticles taken up, the TEM images at areas of 6.25 μm^2^ over a cell from the respective assays were chosen arbitrarily and the microparticles were counted. The same magnification was selected throughout. 50 areas were counted and the means were determined.

### Uptake studies with HUVEC cells

HUVEC cells (Lonza, Basel; Switzerland) were cultured in Endothelial Cell Medium (Gibco/Thermo Fisher Scientific, Waltham, MA) supplemented with 5% heat-inactivated fetal bovine serum (FBS; Gibco/Thermo Fisher), 1% penicillin/streptomycin and vascular endothelial growth factor [VEGF] for rapid proliferation, as described [37]. The cells were cultured at 37°C in humidified 5% CO_2_, 95% air. The polyP preparations (“Na-polyP[Ca^2+^]”, “Ca-P particles” or “Ca-polyP-MP”) were added at a final concentration of 30 μg/mL.

After an incubation period for up to 3 d the cells were stained with the far-red fluorescent DNA dye Draq5 (Biostatus, Shepshed; UK) for nuclear visualization or with DAPI (#D9542; Sigma; 2-(4-amidinophenyl)-6-indolecarbamidine dihydrochloride) for polyP detection [38].

### Microscopic analyses

#### Scanning electron microscopy

To visualize the microparticles in SaOS-2 cells the samples were analyzed by the scanning electron microscope (SEM) ESEM XL-30 (Philips, Eindhoven; Netherlands).

#### Transmission electron microscopy

The ultrathin sections were inspected with a FEI-Tecnai F20 transmission electron microscope (TEM), operated with 200kV (FEI Company, Eindhoven, The Netherlands). The 300–500 nm large polyP microparticles appeared as electron-dense particles in the TEM images; since most of the spherical particles had been sectioned, their diameter appeared around 100 μm.

#### Laser scanning microscopy

To analyze the intracellular accumulation of polyP, the cell samples were inspected by a Zeiss 710 confocal laser scanning microscope (cLSM; Zeiss, Göttingen, Germany), using the excitation lines of a diode laser (405 nm) and a helium/neon laser (633 nm) for DAPI and Draq5 detection respectively. Overlaid image stacks were compiled and computed with the Zen software (Zeiss) using an average projection algorithm. The relative level of polyP within the cells had been normalized to the fluorescence intensity of 658 nm [emission] (Draq5) and is given as the ratio of emission between 480 and 520 nm (DAPI) and 658 nm (Draq5), at the indicated incubation condition [24, 25]. For documentation of the fluorescence by false-color imaging, the Draq5-stained nuclei were visualized in red violet, while the DAPI-stained polyP surrounding the nuclei were visualized in a cloud-like gray-blue color.

### Determination of the intracellular ATP pool

HUVEC cells were used to determine the effect of polyP on the intracellular ATP level. For quantitative ATP-monitoring the luminescence assay was applied; the enzymatic ATP luminescence kit (no. LL-100-1, Kinshiro, Toyo Ink; Japan) was used, as described [4,39–41]. HUVEC cells were cultivated in “Endothelial Cell Medium”/FBS until a density of ≈75% was reached. Then, 30 μg/mL of the respective phosphate/polyP sample were added and incubation was continued for up to 3 h. Where indicated, the cells were pre-incubated with TFP (see above). After having established the standard curve with given ATP, the nucleotide was extracted from the cells and the amount of ATP was estimated (10 independent experiments were performed).

### Statistical analysis

After verification that the respective values follow a standard normal Gaussian distribution and that the variances of the respective groups are equal, the results were statistically evaluated using the independent two-sample Student’s *t*-test [42].

## Results

### Characterization of the particles: XRD, EDX, FTIR spectroscopy and SEM imaging

XRD: The “Ca-polyP-MP” particles were characterized as described before [4]; the 2θ scan showed no distinct diffraction peaks for the “Ca-polyP-MP”, indicating that the particles are amorphous; in contrast, the “Ca-P particles” are crystalline (not shown here).

**Fig 1:**
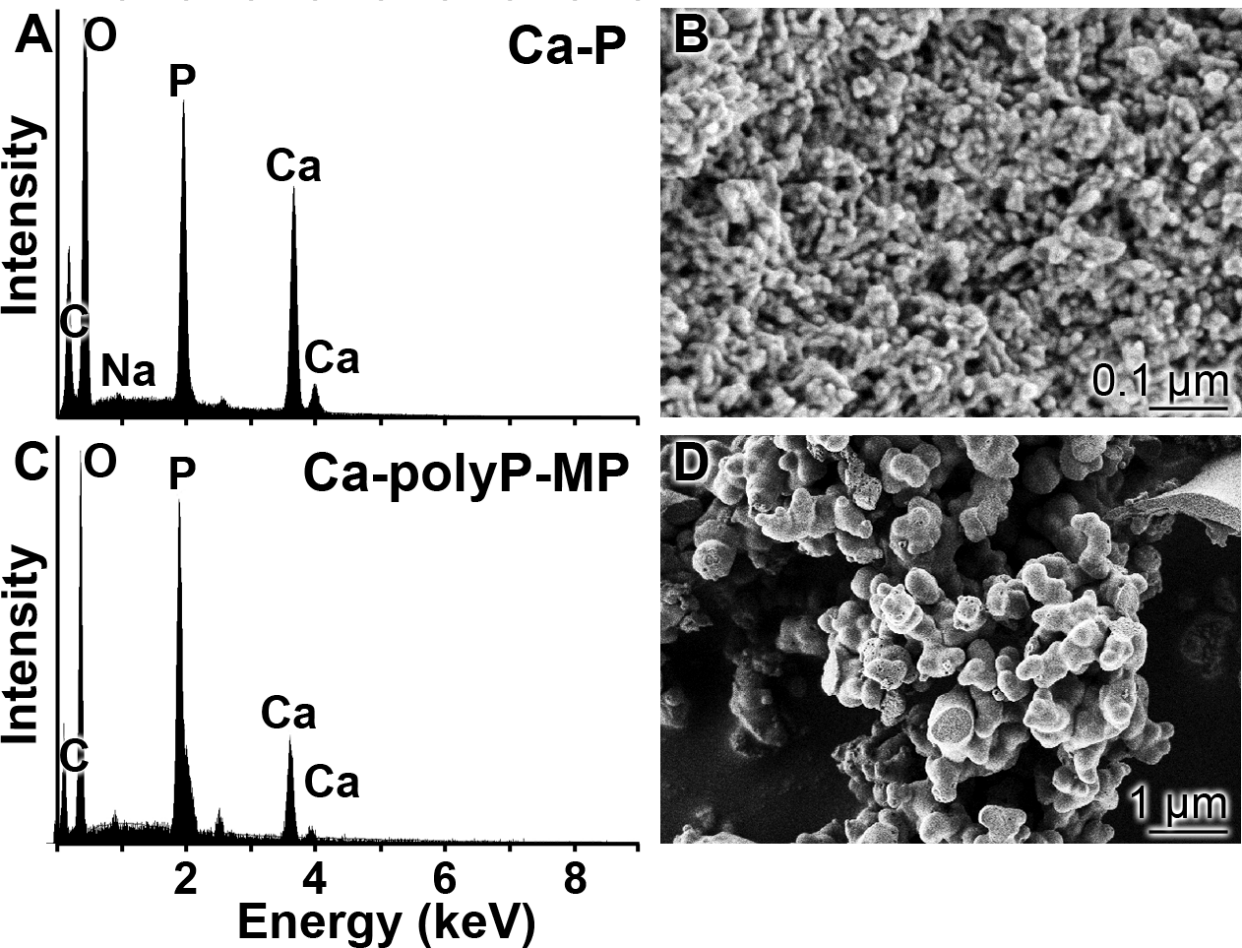
**EDX spectra:** (**A**) “Ca-P particles” and (**C**) “Ca-polyP-MP”. In the right panel the corresponding morphologies, determined by SEM imaging, of (**B**) the “Ca-P particles” and (**D**) the “Ca-polyP-MP” are shown.

EDX: EDX spectra were recorded from samples of “Ca-polyP-MP” and, for comparison purposes, of “Ca-P particles” (Fig 1). The “Ca-polyP-MP” spectrum reveals that the material is composed primarily of Ca, P, and O (Fig 1C). The same elements were recorded from “Ca-P particles” in almost the same abundance (Fig 1A).

**Fig 2:**
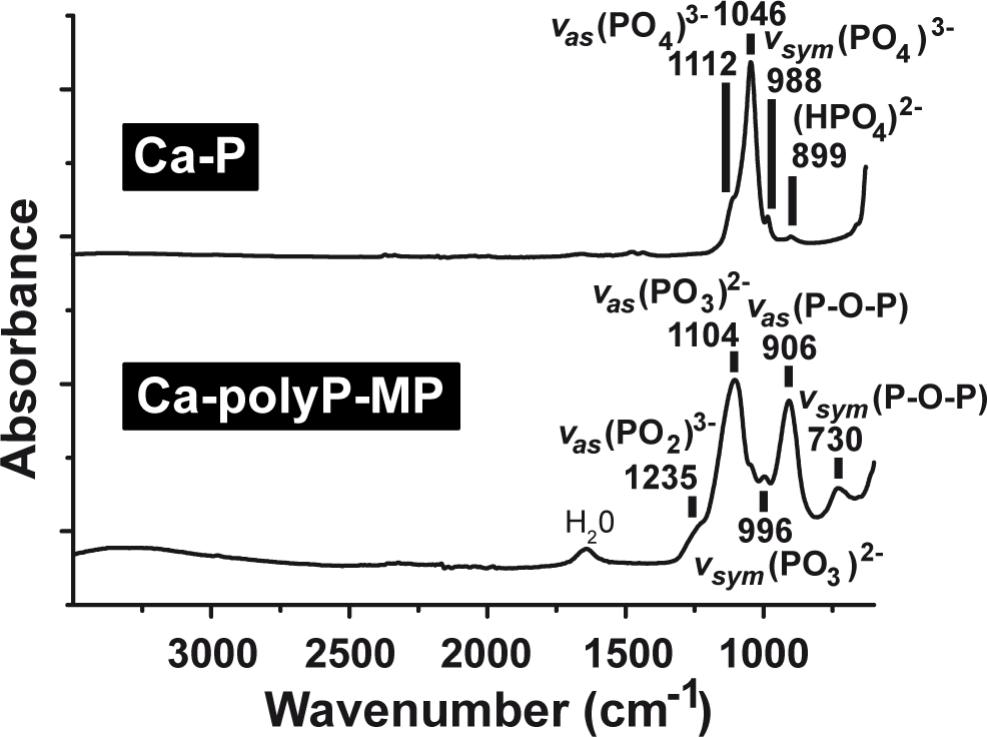
FTIR spectra. The spectra were recorded of “Ca-P particles” (upper panel) and “Ca-polyP-MP” (lower panel) within the range of wavenumbers 3500–600 cm^-1^.

FTIR: As expected for polyP samples, “Ca-polyP-MP” comprises the characteristic asymmetric vibration signals for polyP with a peak near 1235 cm^-1^ indicative for (PO_2_)^3-^ and both the asymmetric and symmetric signal for P-O-P at 906 cm^-1^ and 730 cm^-1^, respectively. The asymmetric and symmetric signal for (PO_3_)^2-^ is found at 1104 cm^-1^ and 996 cm^-1^, respectively (Fig 2). In contrast, the “Ca-P particles” show the characteristic vibration signal at 1046 cm^-1^ (asymmetric) and 988 cm^-1^ (symmetric) for (PO_4_)^3-^ (Fig 2).

SEM: The particle size of the “Ca-polyP-MP” ranges between 300 and 500 nm, as observed by SEM (Fig 1D). The average size of the spherical particles was determined to be 370±85 nm (n = 50). In contrast, the spherical “Ca-P particles” are much smaller with 10–20 nm (average 14±9 nm).

### Effect of *polyP* on the intracellular Ca^2+^ level

**Fig 3:**
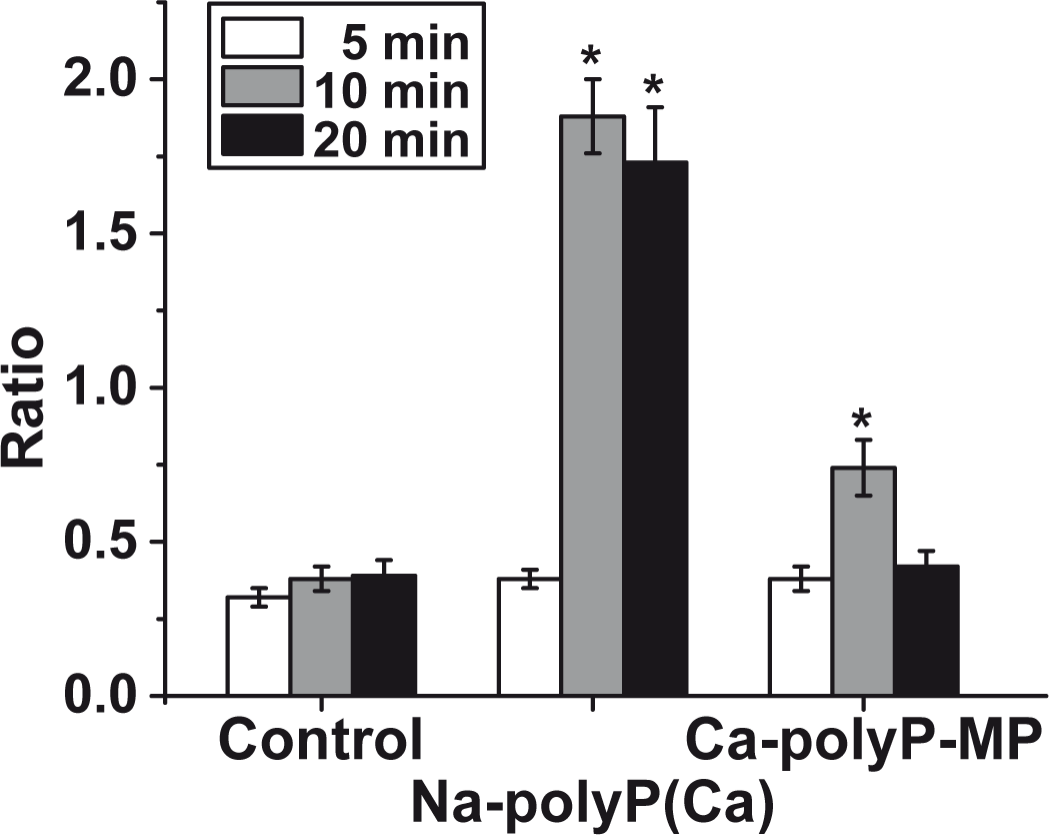
Increase of cytosolic [Ca^2+^]_i_ level upon exposure of HUVEC cells to polyP. The cells were loaded with Fura-2. Subsequently the cells remained either untreated (controls) or were treated with 30 μg/mL of polyP in the soluble form (“Na-polyP[Ca^2+^]”) or the particulate form (“Ca-polyP-MP”). The Ca^2+^ level was assessed by determination of the ratios 340/380 nm. The results are expressed as mean values ± SE; * *p* < 0.05; n = 50.

A change of the intracellular Ca^2+^ level ([Ca^2+^]_i_) causes regulatory effects on eukaryotic protein translation and also a series of cellular ATP-consuming reactions [43]. Previously it has been reported that polyP, applied as “Na-polyP[Ca^2+^]”, causes a distinct and rapid increase of [Ca^2+^]_I_ [26]. Taking Fura-2 as an indicator chemical this effect could be confirmed (Fig 3); exposure of the HUVEC cells to 30 μg/mL of “Na-polyP[Ca^2+^]” results in a rapid increase of the cytosolic [Ca^2+^]_i_ as measured by the 340/380 excitation ratio. After a 10-min incubation period the ratio increased from 0.35 (controls) to 1.88±0.12 units and drops only insignificantly to 1.73±0.18 units after 20 min exposure. In contrast, the particulate polyP, “Ca-polyP-MP”, at a same concentration caused only a light upregulation of the [Ca^2+^]_i_ from 0.35 (controls) to 0.74±0.09 (10 min incubation); after 20 min the signal decreased to a normal level. This finding indicates that polyP microparticles, if taken up by the cells, do not affect intracellular [Ca^2+^]_i_ and Ca^2+^ homeostasis.

### Cellular uptake of polyP microparticles: SaOS-2 cells

**Fig 4:**
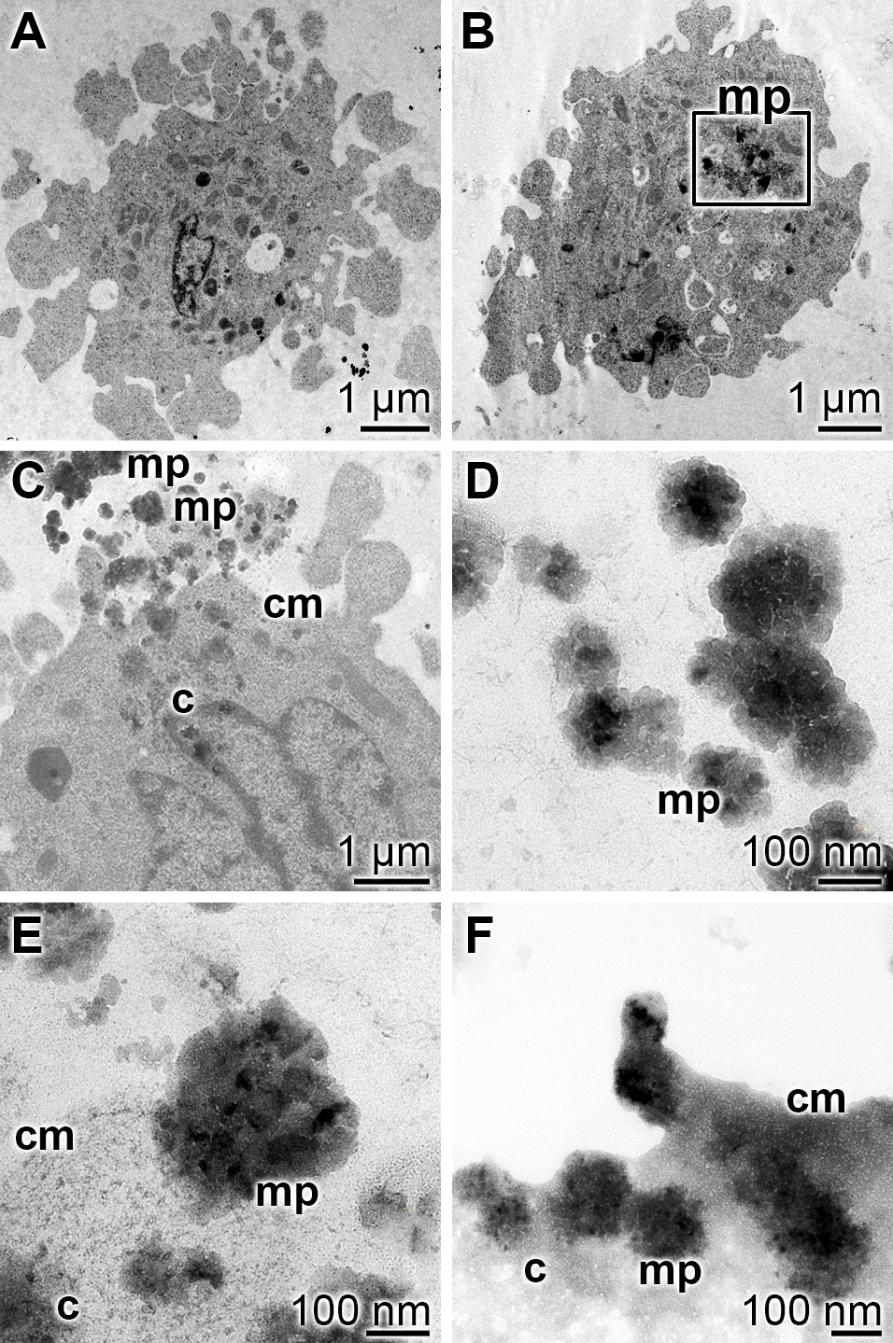
Uptake of microparticles (“Ca-polyP-MP”) by SaOS-2 cells; TEM. The cells were incubated with 30 μg/mL of “Ca-polyP-MP” or remained untreated (controls) for 3 d and then were processed for TEM analysis. The microparticles (mp) can be distinguished from cell constituents by their electron-dense appearance. (**A**) In the control cells no distinct electron-dense particles can be distinguished. (**B** to **F**) In contrast, cells exposed to “Ca-polyP-MP” show abundantly the electron-dense particles; in (B) an area filled with microparticles is highlighted by a square. (C, D) The extracellular applied microparticles associate with the cell membrane (cm) and (E) become internalized into the cell (c). (F) Especially near the cell membrane the particles remain in a distinct shape.

To visualize the uptake of the 300–500 nm large polyP microparticles, “Ca-polyP-MP”, the high resolution F20 FEI-Tecnai TEM was used. For the sake of clarity the uptake studies have been performed with SaOS-2 cells. The cells remained untreated (controls), or were treated with 30 μg/mL of polyP either as soluble “Na-polyP[Ca^2+^]” or as particulate “Ca-polyP-MP” and were incubated for 3 d. Then the cells were fixed and inspected by TEM. It becomes overt that in the controls or in the “Na-polyP[Ca^2+^]” treated cells, no electron-dense particles can be detected; the image of a representative SaOS-2 cell from a “Na-polyP[Ca^2+^]”-treated culture is shown in Fig 4A. However, if the cells are expose to “Ca-polyP-MP” electron-dense microparticles can be visualized (Fig 4B and 4C). The 300–500 nm large particles applied, appear–in the average–as 100 nm large lobulated electron-dense and spherical particles. The smaller size observed was caused by the slicing procedure, with particles sectioned off the equatorial level. The process of uptake of the particles is shown in Fig 4D to 4F. Outside of the cells the particles are freely present (Fig 4D). The particles are taken up into the cells and can be identified relatively easy in the TEM images (Fig 4E), especially if the particles are still in the proximity to the cell surface (Fig 4F). Occasionally the particles are surrounded by clathrin-coated pits [44].

In order to clarify if the observed uptake is dependent on cell metabolism, the cell assays with Ca-polyP-MP were kept at 4°C for 3 d. Then the cells were processed for TEM analysis after slicing. The inspection revealed that no particles accumulated intracellularly (data not shown).

**Fig 5:**
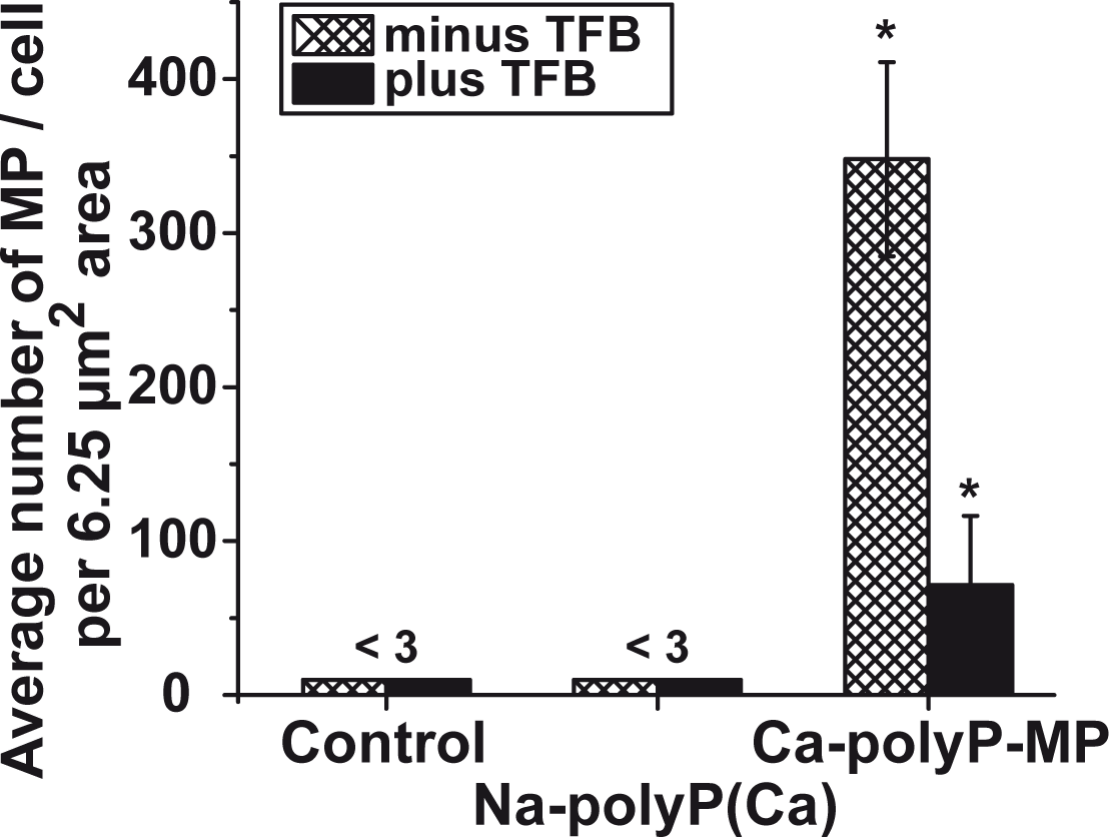
Semiquantitative analysis of the number of particles taken up by SaOS-2 cells. As described under “Material and Methods” the cells were incubated with 30 μg/mL of “Na-polyP[Ca^2+^]” or “Ca-polyP-MP”; the controls remained without polyP. After incubation, the cells were sliced and inspected by TEM. The number of particles per defined cell area (2.5 μm x 2.5 μm; 6.25 μm^2^) after sectioning was determined; mean values ± SE; * *p* < 0.05; n = 50.

To determine semiquantitatively the process of endocytosis, the number of particles taken up by the cells was assessed by counting the microparticles, present in a defined area of 25 μm^2^ over a given cell. The data showed that in cells that remained untreated or were treated with soluble “Na-polyP[Ca^2+^]” no particles can be distinguished in an unambiguous way (Fig 5). However, if cells, incubated with “Ca-polyP-MP” were inspected, very frequently microparticles are seen; the counting revealed 348±63 particles per 6.25 μm^2^ cell area. If those cells were pretreated first with TFP, an inhibitor of clathrin-dependent endocytosis, and subsequently incubated with “Ca-polyP-MP”, only a few particles are seen (65±41 particles per cell).

### Cellular uptake of polyP microparticles: HUVEC cells

**Fig 6:**
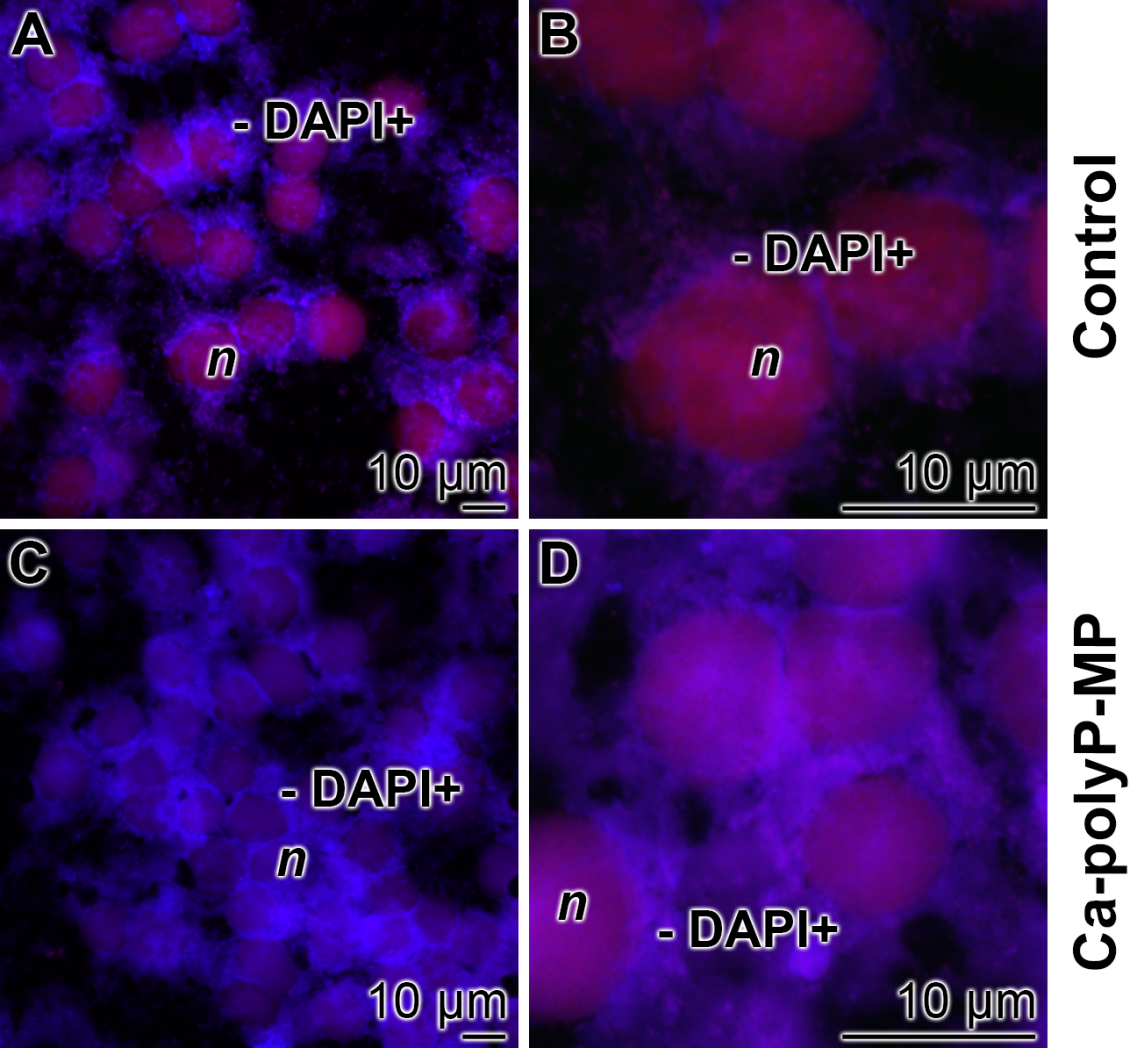
Subcellular localization of DRAQ5-positively stained areas in red fluorescence (nuclei; *n*) as well as DAPI blue fluorescing cytoplasmic regions (DAPI+) in HUVEC cells; cLSM. (**A**, **B**) Controls, not exposed to polyP microparticles; only narrow DAPI+ areas around the nuclei are visible. (**C**, **D**) Cells incubated with 30 μg/mL of “Ca-polyP-MP” for a period of 24 h show a bulky, more homogeneous staining with DAPI of the extra-nuclear environment.

A very convenient approach to assess the intracellular polyP level in HUVEC cells is to stain the cells with DAPI and subsequently visualize the intracellular polyP by cLSM. The polymer accumulates in the cytosol as a blue-whitish cloud, close to the nuclear membranes. In the absence of the polymer the non-homogeneous flocculent rim is comparably small, consisiting of a narrow layer adjacent to the nuclear membranes (Fig 6A and 6B). In contrast, if HUVEC cells were exposed to “Ca-polyP-MP” an expansive area surrounding the nucleus is densly and in most areas almost homogeneously stained (Fig 6C and 6D).

**Fig 7:**
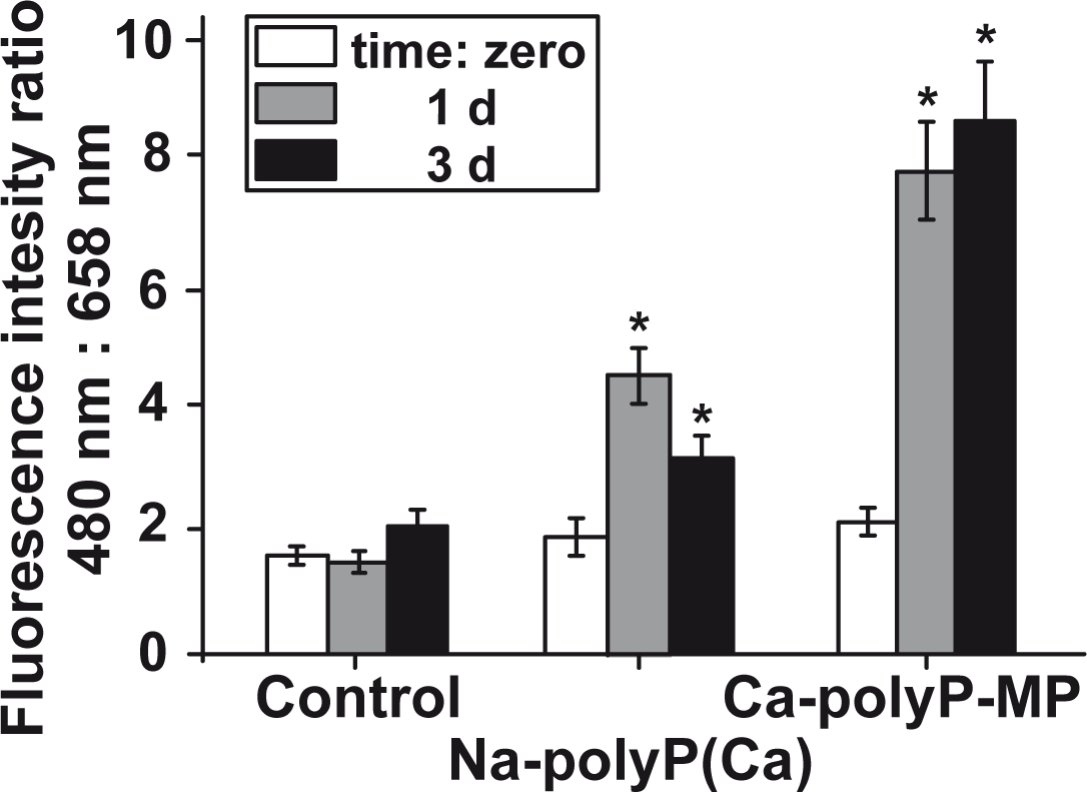
Semi-quantitative analysis of the intracellular accumulation of polyP in HUVEC cells after exposure of the cells to polyP; staining and cLSM analysis. In the control series, no polymer was added to the cells, while in the treated cultures either “Na-polyP[Ca^2+^]” or “Ca-polyP-MP” was added at a concentration of 30 μg/mL. The cLSM analyses have been conducted at time zero, after 1 d or 3 d. Then the intensity ratios between 480 nm (DAPI) and 658 nm [emission] (Draq5) were computed. The mean values ± SE have been determined form 50 parallel measurements; * *p* < 0.05; n = 50.

In a semiquantitative approach to assess the amount of polyP accumulating in HUVEC cells after incubation with the two polyP forms, the intensity ratios between 480 nm (DAPI) and 658 nm (DRAQ5) were determined in the three-dimensional overlay image stacks (Fig 7). At time zero, ratio values in the controls (not treated with polyP) or in the polyP-treated assays, either with “Na-polyP[Ca^2+^]” or with “Ca-polyP-MP” were low, around 1.8 ratio units. Upon incubation of the cells with “Na-polyP[Ca^2+^]” the level of intracellular polyP increases significantly to 4.8 ratio units after 1 d and 3.2 units after 3 d. Much more impressive is the accumulation of polyP in HUVEC cells after exposure to “Ca-polyP-MP”. After 1 d already a level of 7.8 ratio units is reached that increased further after 3 d to 8.4 units.

### Upregulation of the intracellular ATP pool after exposure to “Ca-polyP-MP”

**Fig 8:**
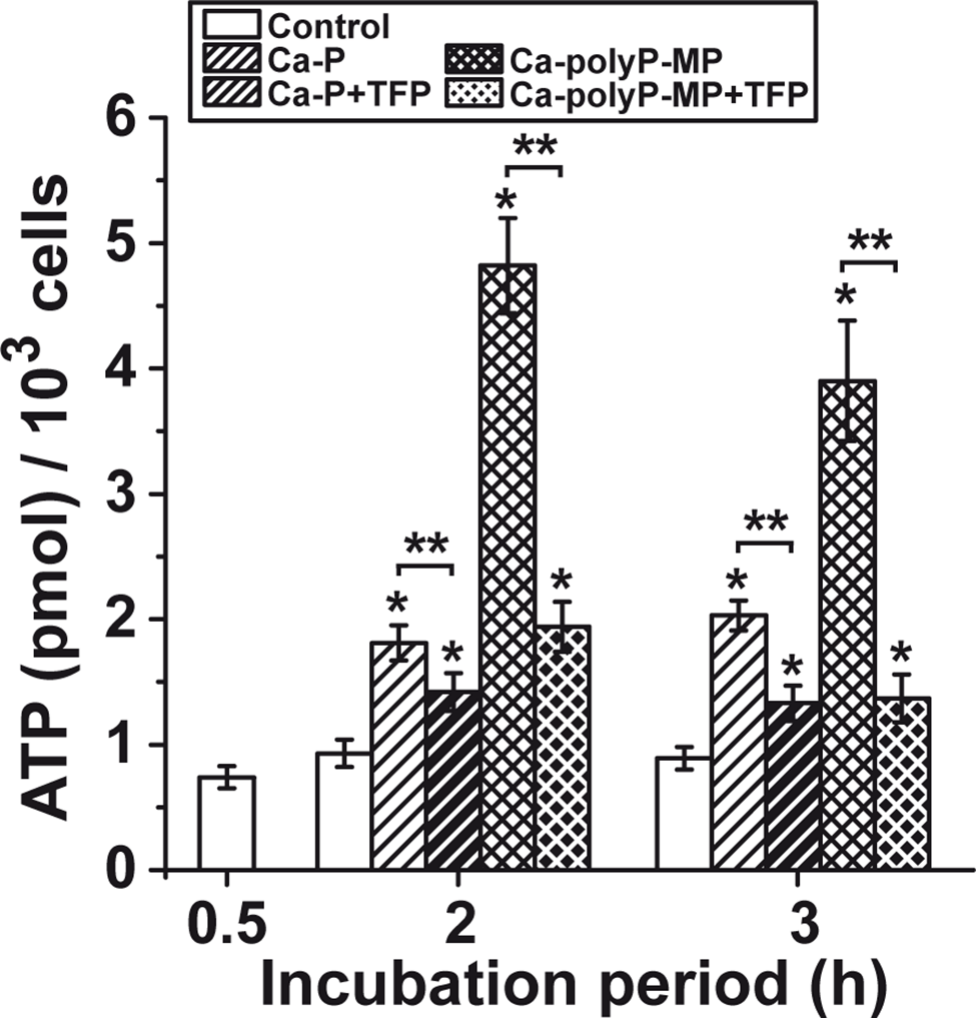
Influence of polyP on the intracellular ATP level. The effects of both “Ca-P particles” [bars hatched to the right] and “Ca-polyP-MP” [cross hatched bars]) are shown; the controls without the polymer were run in parallel [open bars]. The cells were incubated with 30 μg/mL of the particles for up to 3 h; then ATP was extracted and quantitated. In two series of experiments the cells were incubated in addition to phosphate/polyP with 20 μM TFP [bars hatched to the right, white on black; cross hatched bars, white on black]. Significant differences between the control values (minus phosphate/polyP) and the phosphate/polyP test samples are indicated (* *p* < 0.05). In addition, the significance between the controls is indicated (** *p* < 0.05).

The luciferace/luminescence assay was applied to determine the intracellular ATP pool in HUVEC cells. The cells were incubated either with 30 μg/mL of “Ca-P particles” or “Ca-polyP-MP” for a period of up to 3 h. Then the cells were harvested and the extract was prepared. This extract was subjected for quantitative ATP determination. It is seen that, compared to the controls without any phosphate/polyP, in the cell assays with “Ca-P particles” only a moderate raise (90–120% increase) of the intracellular ATP level is measured. In contrast, under otherwise the same conditions the ATP level increased to 510% and 420%, correlated to the controls, in cells incubated with “Ca-polyP-MP” for 2 h or 3 h, respectively. This strong increase is almost completely abolished after pre-incubation of the cells with TFP (Fig 8).

## Discussion

As applied previously, both in *in vitro* [14] and *in vivo* studies [15], we have fabricated also in the present study polyP microparticles, termed as “Ca-polyP-MP”. These particles are amorphous and still contain the polymeric polyP as documented by EDX and FTIR spectroscopy. The average size of the microparticles is 300–500 nm; the particles have a spherical morphology.

The functional signal transduction mechanisms by which polyP microparticles act within HUVEC cells has not yet been described. From previous studies it is known [15] that soluble “Na-polyP[Ca^2+^]” speeds up the process of motility of HUVEC cells leading to faster formation of tubes. At a first sight it was surprising that after exposure of HUVEC cells to particulate polyP microparticles a likewise accelerated pattern formation of cells to well organized 100 to 300 μm sized tubes resulted (data not shown here). In the present report we demonstrate the cellular uptake of polyP microparticles, “Ca-polyP-MP”, and the cellular response to an increased intracellular ATP pool.

In earlier studies it has been described that soluble polyP, administered as “Na-polyP[Ca^2+^]”, increases the intracellular Ca^2+^ level [26]. This observation has been substantiated and extended by claiming that the [Ca^2+^]_i_ is involved in the signaling transduction pathway to apoptosis; however, it should be stressed that this effect is restricted to long chain polyP (> 100 phosphate units), while short and medium sized polyP, like the one used here with an average chain length of 40 phosphate units do not cause this kind of cell death [10]. In the present study the cells were exposed to “Ca-polyP-MP”. The results show that the effect of this microparticulate form of polyP on the intracellular Ca^2+^ level, if compared to soluble polyP (“Na-polyP[Ca^2+^]”), is negligible.

The uptake studies, summarized here, have been conducted with two cell types, i. e. SaOS-2 and HUVEC cells. The SaOS-2 cells have been found to be suitable for the analysis of the uptake process using the high resolution TEM technique. Exposure of these cells to “Ca-polyP-MP” for 3 d showed a distinct accumulation of the particles, especially in the areas adjacent to the cell membrane. The presence of clathrin-coated pits in the vicinity of the particles that are in the process of cellular uptake was not always unambiguous. Therefore, the cells were treated with a non-toxic concentration of TFP, which inhibits dynamin and clathrin-mediated endocytosis [45]. After pre-incubation of the cells with this chemical the uptake of the microparticles was almost completely blocked.

In the second approach HUVEC cells were likewise exposed to polyP formulations. The uptake of polyP and the subsequent accumulation of the intracellular polyP pool had been determined by cLSM after DAPI staining [22–25]. Visual inspection of the cLSM images revealed extensively enhanced intracellular polyP-caused fluorescence emission after exposure to “Ca-polyP-MP”. Moreover, the semi-quantitative assessment, based on the fluorescence ratio of DAPI-stained polyP and Draq5 nuclear staining, revealed a particular strong and time-dependent increase of the intracellular level of polyP for “Ca-polyP-MP”-treated cell samples and a lower rise for the “Na-polyP[Ca^2+^]” samples.

The increase of the intracellular polyP level becomes measurable after 1 d, under the conditions used here. Surprising was the reported finding that the intracellular ATP level increased already after 2 h, in a process that could be almost completely prevented by TFP, an inhibitor of clathrin-dependent endocytosis [35]. This latter result is taken as evidence that the uptake of polyP by the cells and the increase of intracellular ATP are causatively linked processes.

ATP is known to be an important extracellular signaling molecule [46]. Surely ATP is the major intracellular energy source for many physiological and pathophysiological metabolic processes. Furthermore, intracellularly this nucleotide affects allosterically a series of enzymes, such as the AMP-activated protein kinase [47]. However, the role of intracellular fluctuating ATP concentrations and ATP as an intracellular signaling molecule is still largely unknown. The detailed understanding is also hampered by the fact that the intracellular ATP pool is high with 10 mM, in contrast to the corresponding level in the extracellular space, which is approximately 10 nM under basal conditions (reviewed in: [46]). The most dominant intracellular ATP effect is on cytoskeletal dynamics, cellular morphological changes and also intermediary metabolism [48,49].

In addition, the effect on vessel branching of ATP, as a product of glucose oxidation during glycolysis, has been recently studied in detail [50]. Using the HUVEC cell system, we could corroborate that view [15]. These data strongly suggested that not only an intracellular ATP rise but also an exposure to polyP result in an intensified (neo)vascularization. It is a constructive coincidence that almost simultaneously with our report on the acceleration of the wound healing process by application of polyP microparticles [17] an independent report was published that indicated that such a rapid wound regeneration can also be initiated and completed by encapsulated ATP [18]. As main causative factors for this rapid tissue regeneration, both macrophage activation and neo-vascularization had been assumed [18]. In light of the data give here it can be comprehensively and unifyingly been proposed that intracellular ATP, either provided *via* the intermediary metabolic network and/or the mitochondrial oxidation steps coupled with ATP formation or–as proposed here–through metabolic energy transfer from polyP to ATP promote wound healing. The route from ATP to the intracellular proliferation machinery needs to be elucidated. However, at the present stage of knowledge the application of both encapsulated ATP and particulate amorphous polyP should be considered as powerful ingredients for morphogenetically active wound-healing formulations.

## Conclusion

**Fig 9:**
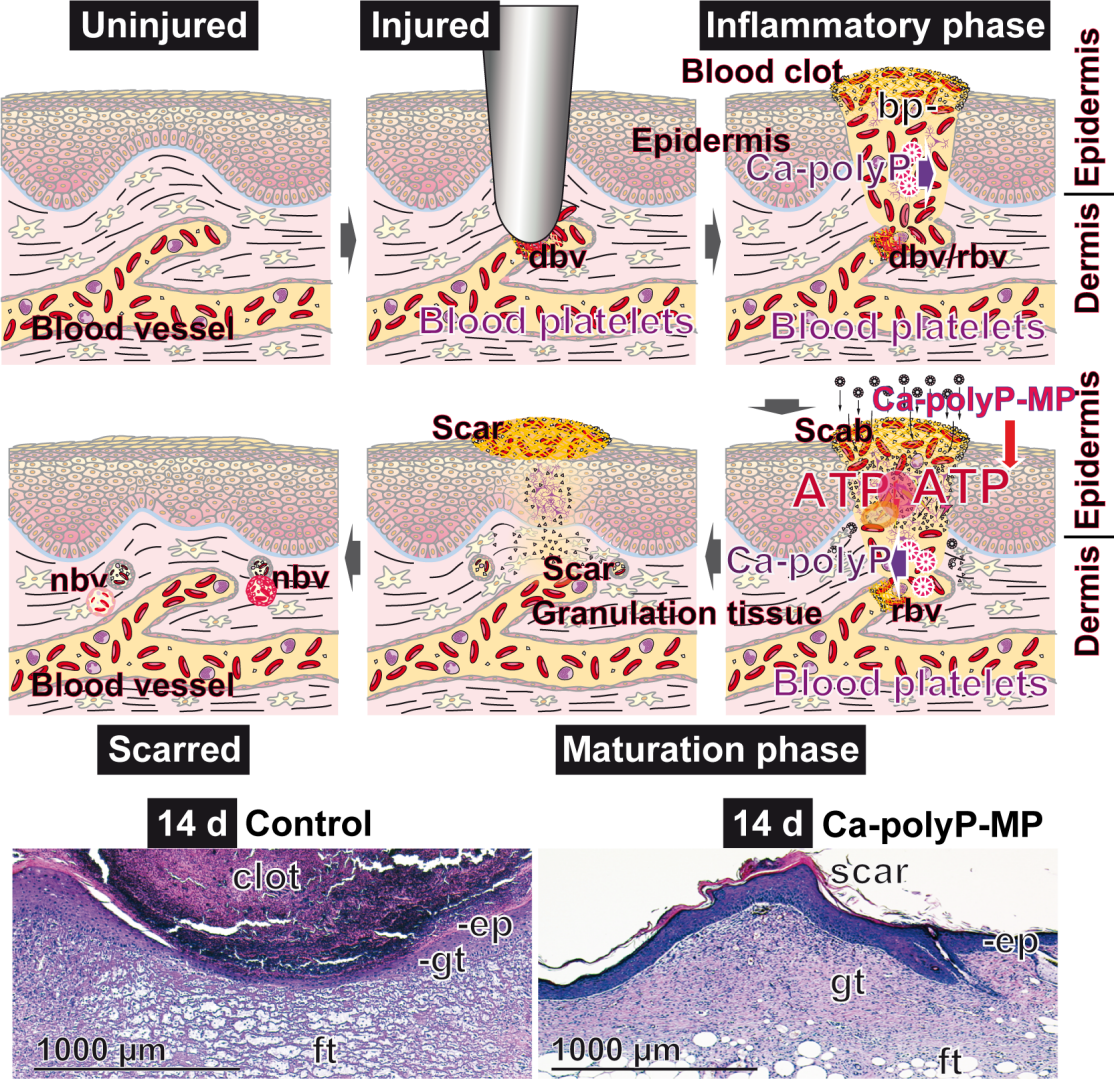
Proposed beneficial effect of exogenously applied “Ca-polyP-MP” on the process of wound healing. (**Upper panel**) During the inflammatory phase endogenous Ca-polyP is released from those blood platelets (bp) that accumulate in the blood clot area. Prior to scab formation, exogenous “Ca-polyP-MP” particles are applied, which are enzymatically hydrolyzed *via* the alkaline phosphatase that is present in the blood plasma. Simultaneously the energy originating from the high-energy phosphoanhydride bonds is transferred to AMP/ADP under formation of ATP. The damaged blood vessel (dbv) system is repaired (rbv) and new blood vessels (nbv) are formed within the granulation tissue in the dermis and epidermis. (**Lower panel**) Representative images are given for a section each through a regenerating wound of an untreated diabetic mouse (left) and a “Ca-polyP-MP”-treated mouse (right) 14 d after setting the wound. A striking difference is seen between these two histological images especially with respect to the bulky clot in the untreated control *versus* the scar in the polyP-treated wound, as well as the presence of a tiny epidermal layer (ep) and simultaneously a thick fat tissue layer (ft) in the untreated control. In the polyP-treated wound both the epidermis (ep) and the granulation tissue (gt) are well developed. The sections are stained with hematoxylin–eosin (to be published).

It is well accepted that (neo)vascularization is a crucial step not only during many physiological tissue repair processes, such as wound healing, but also during tissue modeling/re-modeling under pathological conditions with inflammatory diseases, tumor growth or (diabetic) retinopathy as examples [51]. Focusing on wound healing this complex and dynamic process is characterized by five phases: hemostasis, inflammation, proliferation, remodeling, and finally, re-epithelialization [52]. It is imperative to mention that the first four steps proceed very rapidly within about 3 d [53]. One adverse event seen in delayed wound healing or non-healing wounds is the deficient blood supply [54], turning the wound tissue area to a partially ischemic state [55]. The proposed effect of “Ca-polyP-MP”, administered exogenously is sketched in Fig 9

Based on these two recent directions of studies, i. e. polyP-based treatment [17] and ATP-loaded vesicles [18] of wounds in experimental models, it can be concluded that exogenously administered metabolic energy to wounds, either directly as ATP or indirectly *via* polyP, should be considered as a new promising strategy for acute and chronic wound care.
